# Postural Patterns of the Subjects with Vergence Disorders: Impact of Orthoptic Re-education, a Pilot Study

**DOI:** 10.22599/bioj.116

**Published:** 2018-10-09

**Authors:** Gwenaelle Delfosse, Dominique Brémond-Gignac, Zoï Kapoula

**Affiliations:** 1 IRIS Team – Physiopathologie de la Vision et Motricité Binoculaire – CNRS – Université Paris Descartes, FR; 2 APHP Necker Hospital, FR

**Keywords:** vergence insufficiency, convergence, divergence, orthoptic therapy, postural control

## Abstract

**Aim:**

Vergence insufficiency is a common oculomotor disorder which causes visual but also general, and even postural symptoms. This study aimed to characterise postural control of subjects with isolated vergence disorder and assess whether orthoptic therapy affects it.

**Method:**

Vergence disorders were evaluated and treated by orthoptists. Postural control quality was measured before and after orthoptic therapy in different conditions to study the role of vision, fixating distance, binocular vision and ocular dominance.

**Results:**

Before orthoptic therapy, we recorded less body sway when subjects had their eyes closed than when they had their eyes open, and also less sway for the binocular condition when compared with monocular viewing conditions. This is opposite to well-known normal behaviour. Moreover, no distance or ocular dominance effect was found. After orthoptic therapy, our subject’s body sway was less when they had their eyes open than with their eyes closed and less when they looked at near fixation. No difference was found between monocular and binocular viewing conditions, but a small advantage of ocular dominance was found for one parameter.

**Conclusion:**

We conclude that subjects with vergence disorders show postural behaviour that is not characterized by the normal regularities observed in healthy subjects. Orthoptic re-education may have contributed to promoting such regularities. Further studies are needed to confirm these preliminary results.

## Introduction

Eye movements are not only helpful for vision but also essential for body control and equilibrium. In this study, we demonstrate this concept by examining vergence eye movements. There are several aspects on how viewing conditions influence postural control. Traditional interpretation were in terms of differences in visual feedback, but the recent work of our team emphasised the importance of eye movements and their subtending motor command signals, e.g., converging the eyes via the use of prisms improves postural stability (Lê & Kapoula 2008). There are a number of tests that can be used in research to explore the role of vergence in relation to posture.

*Vision/non-vision – Romberg test:* The Romberg test involves measuring posturography assessments when the eyes are open and when the eyes are closed. The quotient of Romberg is the ratio of posturography values in the eyes-closed condition to the eyes-open condition. The normal quotient of Romberg when fixating a near target is around 2, indicating that postural stability is twice as poor with eyes closed (Lê & Kapoula 2008).

However, the Romberg test, when performed at viewing distances beyond 90 cm, provides a quotient of lower value, close to 1 (Lê & Kapoula 2008); this means that there is no difference between eyes-closed and eyes-opened conditions in postural stability (Lê & Kapoula 2008). As presented by Lê and Kapoula (2008), at such distances the angle of convergence of the eyes is small and is similar in the eyes-open and eyes-closed conditions. In contrast, for closer distance, the convergence angle is higher when the eyes are open and decreases drastically when the eyes are closed. Thus, the Romberg quotient reflected the change in vergence angle with eyes open versus eyes closed that is important when the test is done at near viewing distance but not at far distance.

*Far viewing – near viewing:* When testing posture during natural viewing in different distances, there is a benefit of proximity: postural stability is better when fixating at near than at far (40 cm versus 200 cm). This again is due to the increased convergence angle at near; indeed Kapoula and Lê (2008) have shown that even though when the target fixated is physically at far distance, insertion of convergent prisms over the eyes improve postural stability. Thus, the convergence eye movement *per se* contributes to better postural stabilisation. The benefit of proximity had also been found in dyslexic and non-dyslexic children by Kapoula and Bucci (2007) and in subjects with idiopathic bilateral loss of vestibular function by Kapoula et al. (2013).

*Binocular Vision/Monocular Vision:* Lê and Kapoula (2008) reported no benefit of binocular versus monocular viewing in young and in old subjects. For far vision, the subjects were even less stable with both eyes open than with one eye closed (Lê & Kapoula 2008). Their findings agree with Gentaz’s earlier work, which had shown difference in postural control during monocular viewing. He named “postural eye” the eye that provides better stability (Gentaz 1988). However, Gaertner et al. reported a benefit for bi-ocular vision in strabismic children (who also have vergence disorders) (Gaertner et al. 2013). Bucci et al. (2009) found similar results in children with convergence disorders and vertigo, but only for near fixation. Again the benefit of binocular vision relative to monocular vision was attributed to better control of vergence angle with both eyes open, thereby enabling binocular disparity cues to act; their action would be particularly useful for subjects with strabismus or vergence disorders.

*Ocular dominance:* In accordance with Gentaz’s work, Lê and Kapoula (2006) found smaller oscillation on the anterior-posterior axis with the dominant eye open than with the non-dominant eye open.

Finally, more recent studies comparing fixation at near versus far, i.e., with a greater or smaller vergence angle respectively, with a third condition in which subjects were performed active vergence eye movements between near and far targets, showed a clear benefit of the latter active vergence condition: active vergence eye movements provide better postural control in elderly (Matheron et al. 2016), in patients with vestibular loss (Kapoula et al. 2013) and in strabismus subjects (Gaertner et al. 2013).

To summarise, the existing literature shows that postural stability is better at near with eyes open and converging. Also, it is even better when subjects are making active vergence eye movements (Kapoula et al. 2013; Matheron et al. 2016).

Vergence insufficiency is a common disorder which induces ophthalmic but also general disorders as blurred vision, ocular pain, headaches and tiredness. Orthoptists can prescribe treatment of vergence insufficiency with eye movement exercises such as following a target in space or exposing subjects to retinal disparity methods with the use of stereograms, synoptophore or prisms (Lavrich 2010; Jeanrot, Jeanrot & Spielmann 2011). If vergence movements are essential for postural control, then orthoptic treatment that is expected to modify vergence capacity should translate to some changes of postural control. In this pilot study, we examined the effects of orthoptic rehabilitation of vergence capacity measured by clinical tests on postural control. To our knowledge, there is no previous study on orthoptics and vergence, except from Bucci and Kapoula (2009). That study involved children with a diagnosis of vertigo, whereas here we deal with subjects diagnosed with isolated vergence insufficiency in absence of vertigo. Furthermore, we use accelerometers to evaluate postural behaviour based on the captured sway to the centre of body mass, while in prior studies, podal posturography was used measuring the sway to the centre of body pressure (CoF).

## Method

*Ethics statement:* The investigation complied with the tenets of the Declaration of Helsinki and was approved by the local human experimentation committee, the “Comité de Protection des Personnes” (CPP) Ile de France VI (No: 7,035), Necker Hopital in Paris, France. Written informed consent was obtained from the participants after the nature of the procedure had been explained.

*Subjects:* Nine subjects with vergence insufficiency were enrolled in this study (age range 6–27 years). They were recruited at the hospital’s ophthalmology service during a regular follow-up visit. They had previously had an ophthalmologic and an orthoptic assessment that showed vergence insufficiency without any other ophthalmic disorders, apart from ametropia.

*Clinical characteristics:* Five subjects had optical correction (glasses). With their optical correction, all had visual acuity equal to 10/10 at far (crowded tests of Monoyer Scale, 5 m) and near (crowded tests of Parinaud scale, 40 cm) and stereoacuity equal to 40″ using the Titmus stereo test. All subjects had fusional convergence (measured with prisms) lower than 20Δ at far distance and lower than 35Δ at near distance; four of them had fusional divergence lower than 4Δ at far distance and three had fusional divergence lower than 8Δ. All these values were below the normative values (Von Noorden 2002). Three of the subjects were eliminated for aberrant postural parameters or because they didn’t adhere to the orthoptic training prescribed.

*Orthoptic re-education:* The six remaining subjects followed 12 sessions of orthoptic re-education which lasted approximately 15–20 minutes each at Necker Hospital. Orthoptic assessments were carried out before and after the 12 sessions to evaluate the change of vergence capacity. The average frequency of therapy sessions was one session per week. Posturography tests were done together with the first and the last orthoptic examination after the 12 sessions of vergence training. The completed time window was two to six months between the first session and the last.

Postural recordings: Each postural condition (dominant eye fixing, non-dominant eye fixing, binocular, far eyes open, near eyes open, far eyes closed, near eyes closed) lasted 30 seconds. Subjects wore their own glasses (if worn), and an eye cover around their neck. This was used for the non-vision and for the monocular viewing conditions. Postural body oscillations were recorded while standing, with arms along the body, feet apart to shoulder, barefoot or in socks. All unnecessary items or clothing (jackets, coats, items in the pockets) had to be removed before recording. The size and weight of the subject was recorded.

**Figure 1:**
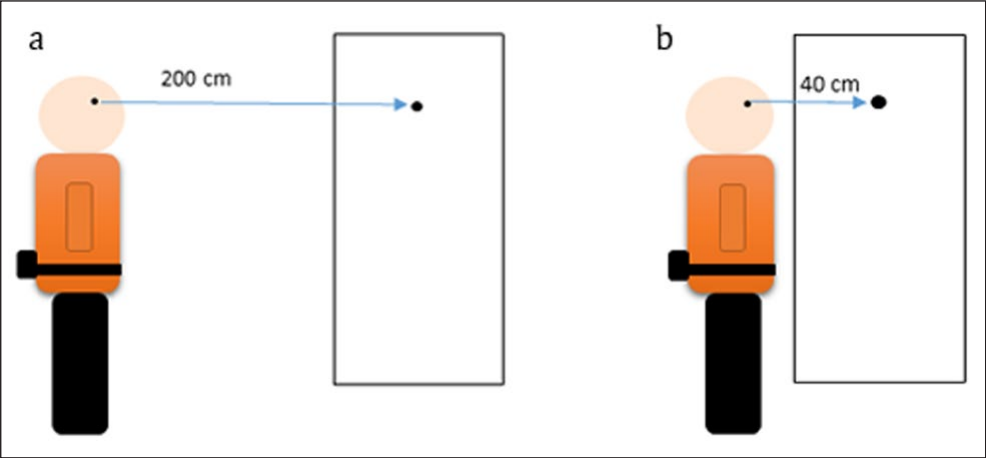
Experimented conditions : the subject who wears the DynaPort looks at the target at far and near distance.

The body sway was measured with the DynaPort® (McRoberts B.V. The Hague, The Netherlands) device (74) equipped with three orthogonally mounted accelerometers in transverse, sagittal and coronal planes (AXXL202, Analogue Devices, Norwood, MA, USA), placed at the lumbosacral level on a belt and near the body’s centre of mass. The sampling frequency is set to 100 Hz. Figure 1a and b illustrates the experimented conditions.

We measured the following parameters: the normalized area (in mm^2^/s), area of an ellipse that contains 95% of the data points, divided by the duration of the measurement, the Root-Mean-Square of Medio-Lateral body sway (RMS of M/L in mm), the Root-Mean-Square of Antero–Posterior body sway (RMS of A/P in mm), the RMS of M/L velocity (in mm/s), the RMS of A/P velocity. These parameters are extracted from the raw gravitational acceleration data in g (1 g ≈ 9,81 m/s^2^). After the data were high-pass filtered, velocity was calculated in the A/P and M/L direction by integration of the acceleration signal and displacement by integration of the velocity signal. The following tests were run:

1. *Romberg test:* The subject viewed a visual target (black dot) placed on a neutral wall at 200 cm from the eyes for far-vision tests (FV) and 40 cm for near-vision test (NV). To test the posture in the absence of vision, the subject closed their eyes after setting the target at far and then at near.
2. *Distance test:* Postural recording was done while the subject look at the target at far distance (200 cm) and then at near (40 cm).
3. *Binocular-monocular test:* The subjects looked at the near target with both eyes open, with an eye cover on his right eye and with an eye cover on his left eye. Results of this test were analysed in term of eye (left versus right) and in term of eye dominance.

### Analysis of data

The statistics software *Statistica*® was used to analyse the parameters. Given the limited number of subjects, non-parametric statistics were used. The Wilcoxon test was used to evaluate difference between before and after orthoptic rehabilitation.

## Results

### Modification of vergence capacity after orthoptic rehabilitation

We noted a modification of vergence capacity after orthoptic re-education. Before orthoptic re-education, all of our subjects had poor convergence capacity, but four of them also had poor divergence capacity. This population is quite representative of what we can see in clinical practice. There was a substantial increase of convergence amplitude measured with prisms, i.e., an average increase of 30Δ for near vision and an increase of 19.5Δ for far vision; moderate but significant increase for divergence capacity, 5Δ for near vision and an increase of 3.7Δ for far vision. All subjects had good stereoacuity (40″). This indicated that our population is a healthy population with normal binocular vision except for their vergence capacity that was weak before training.

### Modification of postural behaviour after orthoptic re-education

#### Romberg test

**Figure 2:**
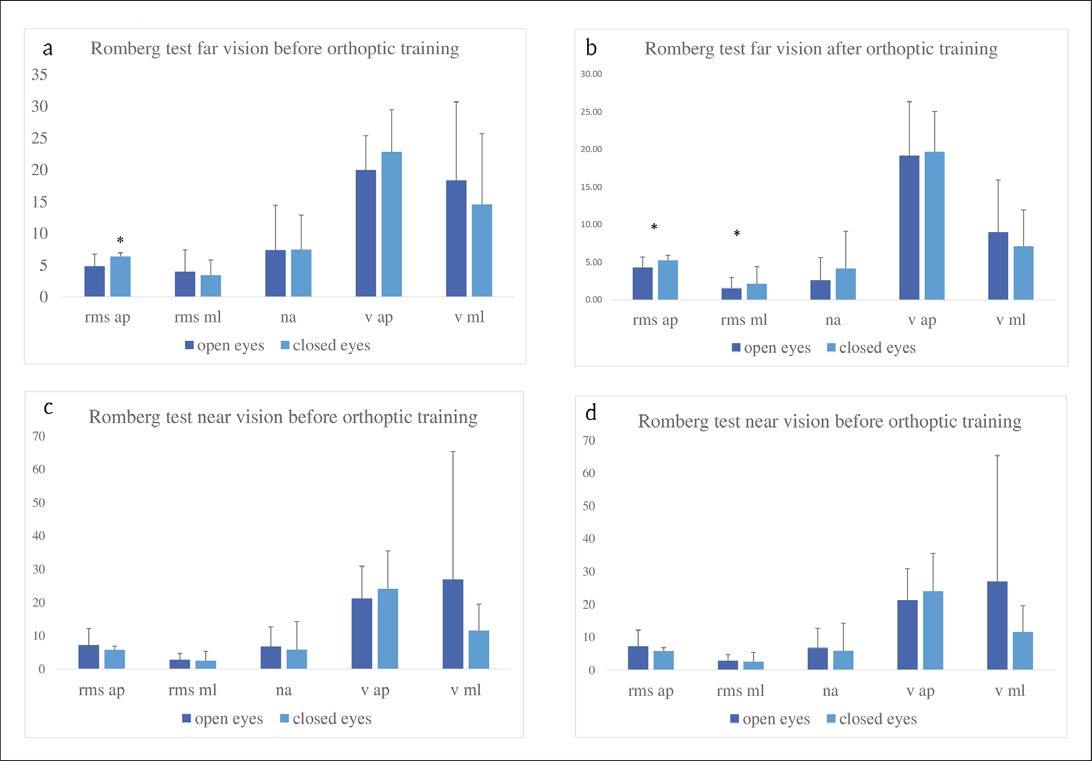
Group means and standard deviations for each postural parameter: Root Mean Square of anteroposterior (RMS ap), for mediolateral (RMS ml) body sway, the Normalized Area (NA), the Velocity of anteroposterior (V ap) and of Mediolateral (V ml) body sway. The values are shown for far vision (**a, b**) and for near vision (**c, d**) before and after orthoptic re-education. In each group are shown the values for open- versus closed-eyes postural condition. Statistically significant differences between eyes-closed and eyes-open conditions are indicated by an asterisk.

Figure 2a and b show that before orthoptic re-education, vision was not helpful for postural control at far or near distance. Indeed, there was no significant difference between posture with open eyes and closed eyes for either viewing distance, except for the anteroposterior body sway at far vision (Wilcoxon Z = 1.99, p = 0.046).

Figure 2c and d show the results after orthoptic re-education. The anteroposterior and the mediolateral body oscillations were higher with closed eyes than with open eyes for far vision (RMS ap Z = 1.99, p < 0,047; RMS ml (Z = 2.20, p < 0.0278)). For near vision, the anteroposterior body sway velocity with closed eyes was significantly higher (V ap Z = 2.20, p < 0.0278). After orthoptic re-education there were some indications for better postural control with open eyes than with closed eyes.

#### Distance effect

**Figure 3:**
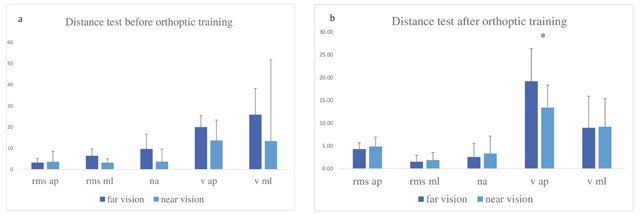
Group means and standard deviations for each postural parameter for near vision versus far vision before (**a**) and after (**b**) orthoptic re-education. All other notations as in Figure 2.

The results are shown in Figure 3a and b before orthoptic re-education. We observed no significant difference between body oscillation parameters at far vision or at near vision. After orthoptic re-education (Figure 3b), the velocity of the anteroposterior sway decreased significantly (Vap Z = 2.20, p = 0.0278).

#### Binocular vision effect

**Figure 4:**
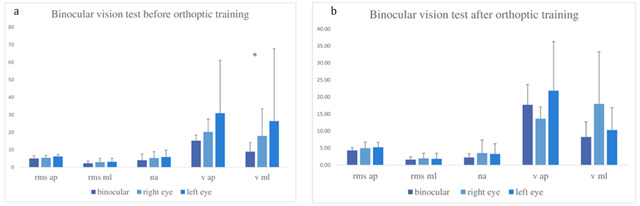
Group means and standard deviations for each postural parameter for binocular vision versus right-eye viewing and left-eye viewing before (**a**) and after (**b**) orthoptic re-education. All other notations as in Figure 2.

Figure 4a shows that the body oscillations on the mediolateral axis were slower when both eyes were open than when the right eye was open (Vml Z = 1.99, p = 0.0464) or when the left eye was open (Vml Z = 1.36, p < 0.0465) before orthoptic treatment. After orthoptic re-education, there was some indication for better postural control at near vision. After reduction, we found no difference between the three conditions (Figure 4b). The advantage of binocular vision versus monocular vision disappeared after orthoptic re-education.

#### Ocular dominance

**Figure 5:**
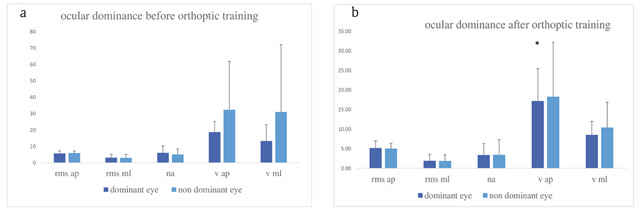
Group means and standard deviations for each postural parameter for dominant-eye viewing versus non-dominant-eye viewing before (**a**) and after (**b**) orthoptic re-education. All other notations as in Figure 2. Comparisons were done for each condition and for each of the parameters of the results shown in Figures 2, 3, 4, and 5. Overall the values were not statistically different between the two testing sessions that were separated by two to six months. There were two significant improvements: the mediolateral body sway with eyes open at far distance and the normalized area under the same conditions decreased after orthoptic re-education.

Results are shown in Figure 5a and b before orthoptic re-education. No difference was observed on body sway between dominant and non-dominant eye-open testing condition (Figure 5a). After orthoptic re-education, we found slower velocity of anteroposterior sway when viewing with the dominant eye (V ap Z = 2.20, p < 0.02778). Wilcoxon test showed that the body oscillation characteristics are significantly different between before and after orthoptic re-education at far distance when both eyes were open (RMS ml Z = 1.99, p < 0.047; NA Z = 2.20, p < 0.028). The same result was found when both eyes were closed for the anteroposterior axis (RMS ap Z = 2.20, p < 0.0278).

In summary, following orthoptic re-education some of the well-known regularities reported in the literature for normal subjects were observed in our patient cohort: the emergence of better control was found with eyes open when fixing at near and no differences between binocular and monocular viewing.

## Discussion

Vergence capacity were measured before and after 12 sessions of orthoptic re-education. The results of our study are important. Firstly, they confirm what is already known in the literature on postural control for healthy subjects. Secondly, they demonstrate the impact orthoptic re-education has on postural control. Orthoptic rehabilitation did increase the vergence capacity both for convergence and divergence.

### Abnormalities of posture before re-education

Before orthoptic re-education several typical aspects of normal postural control were not present in our population of young subjects with isolated vergence insufficiency. Namely, better postural control with eyes open than with eyes closed was absent; this is well documented in healthy subjects (Lê & Kapoula 2008). In our population, the benefit of proximity, i.e., better postural control at near vision than at far vision, is well documented for healthy subjects (Lê & Kapoula 2008; Lê & Kapoula 2006) but was absent in our study participants. Finally, the subjects studied here were more stable with both eyes open than with one eye open.

All these elements suggest a deficit in proper control of the body oscillations, perhaps due to reduced muscular tones in orthostatic position. We attribute this inefficiency to the weakness of the vergence eye movement signals to our subjects. Inappropriate vergence angle control could cause instable vision and this could interfere with the quality of postural control. Similarly, the reduced capacity of convergence did not enable improvement of postural control at near vision relative to far vision because vergence control, despite its insufficiency, could still be better with both eyes viewing than with one eye viewing.

### Postural control after orthoptic re-education

Even though the postural values did not change greatly between the two sessions (separated by two to six months) there were important changes in terms of the Romberg test of the distance effect and of the binocular vision effect. After orthoptic re-education, some characteristics of normal postural control described in the literature became present in our subjects. We observed a better postural control with both eyes open than with closed eyes in terms of mediolateral and anteroposterior body sway. Moreover, the velocity of the anterior posterior body sway decreased at near vision relative to far vision. Finally, we also observed a moderate advantage of the ocular dominance for the velocity of anteroposterior body sway.

All these benefits could be mediated by the improvement of the vergence eye movement signals mediated by orthoptic re-education. Although there is no objective measurement of vergence eye movements in this study, there is evidence in the literature that orthoptic re-education improves some aspects of eye movements, namely their latency. Bucci et al. (2004) have shown a decrease of eye movement latency following two sessions of orthoptic re-education, but no changes in the dynamics of vergence (its speed and duration). It is possible that this benefit in latency occurred in the present study as well. Shortening vergence latency could help better integration of visual vestibular and somesthetic subtending postural control. This interpretation is in line with prior studies showing that vergence eye movements are important for postural control (Gaertner et al. 2013; Matheron et al. 2016).

Indeed, an improvement of vergence capacities as measured by the orthoptist with a prism bar reflects what the subjects do in everyday life, converge or diverge their eyes and adjust the appropriate vergence angle better. Talasan and al. (2016) showed that orthoptic re-education leads to improvements of vergence eye movements measured with video oculography. On the other hand, Kapoula et al. showed that vergence eye movements help to improve postural control in several populations: in dyslexic children (Kapoula & Bucci 2007), in patients with vestibular loss (Kapoula et al. 2013), and in strabismic children (Gaertner et al. 2013).

This improvement of vergence mediated by orthoptic re-education could be at the origin of postural improvements, in terms of finding regularities similar to those seen in normal subjects (e.g., better control when both eyes are open for near vision). The paradoxical result that the postural benefit in favour of binocular versus monocular vision disappeared after orthoptic re-education is also in line with known behaviour of healthy subjects. Indeed, healthy subjects do not show systematically better control with binocular viewing than with monocular viewing (Lê & Kapoula 2008), but strabismic children do (Gaertner et al. 2013). After orthoptic re-education, subjects behaved more like ‘normal’ subjects (i.e., individuals with no vergence anomalies). We hypothesise that with orthoptic re-education, vergence control improves under both monocular and binocular viewing and that the binocular benefit is not systematic anymore.

In conclusion, this study demonstrates that subjects with isolated vergence insufficiency have some postural abnormalities, e.g., lack of known postural regularities. Secondly, orthoptic re-education allows restoration of some aspects of such regularities. This study is the only study that has examined vergence insufficiency alone. Bucci and al. (2009) examined children recruited at the ENT service because of vertigo associated with vergence abnormalities. Furthermore, our study is the first using an accelerometer measuring the centre of body mass, while all previous studies (except that of Matheron who used a platform of feet posturography to measure the centre of body pressure) (Matheron et al. 2016). Although the two measurements do correlate – podal posturography measures the centre of body pressure, whilst the accelerometer measures at the lumbosacral level the body’s centre of mass – the methods and techniques are not identical.

The results of our study suggest that orthoptic re-education can improve postural control. However, it should be noted that a limitation of the study is an absence of a control group who did not undergo orthoptic training. In addition, the small number of study participants should be acknowledged. This preliminary study opens the field for further multisensory investigations combining measures of quality of vergence eye movements and of postural control in the evaluation of vergence insufficiency and its orthoptic treatment. Such an approach has been recently developed by our laboratory (Morize & Kapoula 2017). Novel technologies, based on research ocular motor adaptation, are needed to induce vergence efficiency, including normalisation of its velocity and duration. Such studies could include a comparison of subjects with symptoms (e.g., vergence accommodation disorders) versus healthy subjects to substantiate the pathophysiology of vergence disorders and its neurorehabilitation.

## Additional File

The additional file for this article can be found as follows:

10.22599/bioj.116.s1 Clinical charateristics. Table with vergence values, optical correction and dominant eye for each subject.
