# Supplementary material for: Postural Patterns of the Subjects with Vergence Disorders: Impact of Orthoptic Re-education, a Pilot Study
Source: Br Ir Orthopt J. 2018 Oct 9;14(1):64–70. doi: 10.22599/bioj.116 (PMC7510372; doi:10.22599/bioj.116)
Supplement: Clinical charateristics. — Table with vergence values, optical correction and dominant eye for each subject. [file bioj-14-1-116-s1.pdf]

Subjects (age)	Treatment (time in mouth)	Optical correction (d)	Dominant Eye	Divergence (pD)				Convergence (pD)			
		Right		Before		After		Before		After	
		Left		Far	Near	Far	Near	Far	Near	Far	Near
A (7)	orthoptic training + optical correction (3)	+0,75<br>+0,75	L	**2**	**4**	6	8	**6**	**14**	25	30
B (10)	orthoptic training + optical correction (3)	+ 1,25 (-0,75) 5°<br>+1,25 (-1,25) 0°	L	4	10	8	12	**16**	**25**	30	40
C (11)	orthoptic training + optical correction (3)	+0,75(-0,25) 95°<br>+0,75(-0,25)140°	L	**2**	**6**	6	14	**4**	**14**	25	65
D (23)	orthoptic training (2)	/<br>/	R	**2**	**6**	8	14	**8**	**14**	25	40
E (25)	orthoptic training (6)	(-0,25 à 100°)<br>+0,25 (-0,5) 120°	R	**2**	8	6	14	**6**	**20**	35	60
F (27)	orthoptic training + optical correction (2)	+0,75<br>+1(-0,5)90°	R	8	12	8	14	**12**	**30**	30	60
